# Comparison of the Anxiolytic and Analgesic Effects of Gabapentin and Pregabalin in Cats: A Systematic Review

**DOI:** 10.3390/ani15162346

**Published:** 2025-08-11

**Authors:** Agatha Elisa Miranda-Cortés, María Guadalupe Prado-Ochoa, Roberto Díaz-Torres, Alicia Pamela Pérez-Sánchez, Juan Carlos Del Río-García, Daniel Mota-Rojas, Ismael Hernández-Avalos

**Affiliations:** 1 Program of Doctorado en Ciencias de la Producción y la Salud Animal, Pharmacology and Toxicology of Substances of Veterinary Interest, Universidad Nacional Autónoma de México (UNAM), Cuautitlán 54714, Mexico; 2 Biological Sciences Department, Facultad de Estudios Superiores Cuautitlán, Universidad Nacional Autónoma de México (UNAM), Cuautitlán 54714, Mexico; mcjcrg@cuautitlan.unam.mx; 3 Multidisciplinary Research Unit, Laboratory 9, Cellular Toxicology, Facultad de Estudios Superiores Cuautitlán, Universidad Nacional Autónoma de México (UNAM), Cuautitlán 54714, Mexico; 4 Department of Research and Teaching at the Veterinary Hospital for Dogs and Cats, Speciality in Medicine and Surgery for Dogs and Cats, Universidad Popular Autónoma del Estado de Puebla, Puebla 72410, Mexico; aliciapamela.perez@upaep.mx; 5 Neurophysiology of Pain, Behavior and Animal Welfare Assessment, Department of Agricultural and Animal Production, Universidad Autónoma Metropolitana (UAM), Xochimilco Campus, Mexico City 04960, Mexico; 6 Clinical Pharmacology and Veterinary Anesthesia, Biological Sciences Department, Facultad de Estudios Superiores Cuautitlán, Universidad Nacional Autónoma de México (UNAM), Cuautitlán 54714, Mexico

**Keywords:** gabapentin, pregabalin, stress, fear, anxiety, acute pain, maladaptive pain, cats

## Abstract

The present study aimed to systematically review the anxiolytic and analgesic effects of gabapentin and pregabalin in domestic cats to assess the quality of using these medications for stress, fear, and anxiety management, and the treatment of both acute and chronic pain. The search was carried out between March and May 2025 using four databases: PubMed, Scopus, ScienceDirect, and Google Scholar. The keywords used were the combination of “pregabalin,” “gabapentin,” “analgesia,” “anxiety,” “stress”, and “cats”. Narrative reviews, as well as experimental and observational studies, were included. Experimental studies were classified as randomized controlled trials, controlled clinical trials, and prospective clinical trials, using the GRADE Pro GDP software (FP7-HEALTH.2010.3.1-1-two stage) to assess the certainty and confidence of the evidence. The initial search identified 57 manuscripts, of which 40 met the inclusion criteria. These studies focused on the management of stress, fear, and anxiety, as well as the control of acute and chronic pain in cats. Of these, 21 mention the use of gabapentin as an anxiolytic, while five report similar therapeutic effects of pregabalin. Regarding pain treatment, 12 papers and 2 papers support the use of gabapentin and pregabalin, respectively. This study confirms the validity of both drugs as therapeutic options for the management of stress, fear, and anxiety that impact the emotional welfare of cats. Furthermore, these drugs have been included in therapeutic guidelines for the control and treatment of acute and chronic pain in domestic cats. In conclusion, this systematic review supports the use of both drugs. Still, it highlights the need for more in-depth research and additional clinical trials to complement the existing evidence on the use of gabapentin and pregabalin.

## Simple Summary

The present study aimed to systematically review the anxiolytic and analgesic effects of gabapentin and pregabalin in domestic cats. The search was carried out between March and May 2025 using four databases: PubMed, Scopus, ScienceDirect, and Google Scholar. The keywords used were the combination of “pregabalin,” “gabapentin,” “analgesia,” “anxiety,” “stress”, and “cats.” Narrative reviews, as well as experimental and observational studies, were included. The GRADE Pro GDP program was used to assess the certainty and confidence of the collected evidence. Overall, 40 papers met the inclusion criteria, and 21 papers addressed the use of gabapentin as an anxiolytic, and five reported similar therapeutic effects of pregabalin. Regarding pain management, 12 and two publications report the use of gabapentin and pregabalin, respectively. This study validates both drugs as therapeutic options for managing specific conditions, such as stress, fear, and anxiety, events that affect the emotional welfare of cats. In conclusion, the review supports the use of both drugs, although it highlights the need for additional clinical trials to complement the existing evidence.

## 1. Introduction

Domestic cats are frequently exposed to potentially stressful situations that affect their welfare and overall health. This causes behavioral and habit changes that might affect their interaction with humans and conspecifics. The consequences of these stressful experiences lead to health issues that require veterinary attention for proper evaluation and treatment. Pain is one of the main causes of stress in cats and requires prompt and objective assessment to ensure an adequate analgesic therapy [1,2,3].

Stress is a negative emotion that involves physiological and behavioral changes caused by harmful or unpleasant stimuli. This is also related to the development of negative emotions that are perceived as threatening, whose purpose is self-protection and survival improvement [4]. Particularly, stress in cats has been studied comparatively with other domestic species. However, the responses to stress in domestic felines not only depend on the situation, but also on the temperament of each animal [5].

Cats can also be exposed to stress induced by anesthesia or surgery during different diagnostic procedures or surgical procedures, such as those related to neutering. These modifications activate the hypothalamic–pituitary–adrenal axis and the sympatho-adreno-medullary system, which increases the concentrations of adrenocorticotropic hormone (ACTH) and cortisol [6]. These neuroendocrine alterations are an unconscious response that alters or threatens the animal’s homeostasis and is derived from tissue damage generated by injury or trauma, by the effect of anesthetic drugs, surgical management and even intraoperative nociception. Among the notable modifications are those related to autonomic, metabolic, hormonal, immunological, and neuroendocrine effects [7,8,9,10].

These neuroendocrine alterations lead to changes in the GABAergic system in different cerebral regions and are associated with emotions and reactions during the perception of acute stress. Specifically, the GABAergic system in brain structures such as the amygdala and hippocampus affects stress regulation [11]. These responses can lead to dysfunction in the prefrontal cortex, resulting in an inability to inhibit amygdala activity or overstimulation of the amygdala, which causes fear or stress responses that are both persistent and disproportionate. This dysregulation impairs cognitive flexibility, limiting an individual’s ability to learn from new experiences without fear and fostering avoidance behaviors [12].

Pharmacotherapy significantly reduces these brain modifications and the cat’s protective emotional bias, as well as the potential distress, without replacing the mechanism to respond to distress during feline interactions, so they should be used simultaneously when indicated [4]. In this context, it has been suggested that using stress reduction protocols with facial pheromones or pharmacological treatments such as gabapentin and other anxiolytics may lead to more cat-friendly handling. This approach decreases sedation time and reduces the need for propofol during anesthetic induction. As a result, the findings indicate that preoperative stress reduction protocols could positively influence perioperative outcomes (especially in the trans-anesthetic and trans-surgical) [13].

The therapeutic use of gabapentinoids for the management of postoperative pain in veterinary patients is a topic that is still under development. In this regard, Steagall [2] and Steagall et al. [14] mention that postoperative analgesia produced by gabapentin (using 50 mg, PO, 12 and 1 h before surgery) plus buprenorphine was similar to the combination of meloxicam with buprenorphine in cats undergoing ovariohysterectomy, suggesting that gabapentin may be a therapeutic option for postoperative analgesia, given that the results of acute pain assessment scores and the use of rescue analgesics did not present significant differences between treatment groups. Therefore, the evidence shows that gabapentin is effectively associated with pain control and a decrease in the requirement for postoperative analgesics. Studies have reported their inhibitory properties by interacting with voltage-gated calcium (Ca^2+^) channels in the α2δ−1 subunit. After nerve injury, the α1 subunits of these channels, predominantly of the N type, translocate from the cytoplasm to the cell membrane in the presynaptic terminals of dorsal horn ganglion neurons. These gabapentinoids also inhibit this transport in the presynaptic terminal, blocking the anterograde progression of this process through the axonal cytoplasm. In addition, other mechanisms have been proposed, such as interaction with N-methyl-D-aspartate (NMDA) receptors and transient potential channels, which could contribute to the reduction in neuropathic pain. Modulation of supraspinal noradrenergic receptors, as well as influence on cytokines and protein kinase C, have also been associated with the administration of these drugs. The implementation of multimodal perioperative analgesia prevents sensitization of peripheral and central inflammatory response systems to trauma and surgical stress [15,16].

This systematic review was conducted according to the PRISMA guidelines [17], with the following aims: (a) to evaluate and to compare the anxiolytic and analgesic efficacy of gabapentinoids in domestic felines, and (b) to evaluate the quality of the evidence associated with the use of these drugs in specific contexts, to formulate recommendations for future research.

## 2. Materials and Methods

### 2.1. Search Strategy and Criteria

The search was conducted in the PubMed, Scopus, ScienceDirect, and Google Scholar databases, accessed between March and May 2025. Keyword combinations included “pregabalin,” “gabapentin,” “analgesia,” “anxiety,” “stress,” and “cats.” After removing duplicate studies, eligible studies were manually selected by reviewing titles, abstracts, and full articles. The study selection method was “Record Assessment by More Than One Reviewer” to ensure a more reliable approach than a review.

According to the PRISMA guidelines (www.prisma-statement.org, accessed on 20 February 2025), the selected studies were imported into EndNote, where duplicate manuscripts were removed. The primary reviewer conducted a thorough assessment of the relevance of all titles and abstracts for subsequent selection. In cases of disagreement during the full-text review, a second reviewer assumed responsibility for reviewing the manuscripts and subsequently making the final decision on inclusion or exclusion of the study. The inter-rater reliability was determined using Cohen’s Kappa method, which was 0.78, demonstrating substantial agreement between evaluators.

### 2.2. Inclusion Criteria

The selected peer-reviewed manuscripts were written in English and published between 2015 and 2025. Selected papers used gabapentinoids (gabapentin and pregabalin) to treat stress and anxiety in felines, as well as their application as analgesic adjuvants for the management of acute and chronic pain.

### 2.3. Exclusion Criteria

Abstract-only and commentary papers were excluded. Publications that did not analyze the anxiolytic or analgesic effects of gabapentinoids were also excluded. Studies conducted in other species and clinical case reports in which pregabalin or gabapentin were mentioned solely as part of a treatment without analyzing the results were also excluded.

### 2.4. Classification of Eligible Studies

Selected studies were classified as follows:

- Gabapentinoids: gabapentin and pregabalin in felines.
- Anxiolytic effects of gabapentinoids in cats.
- Gabapentin and pregabalin as treatment for acute and chronic pain in cats.

The certainty and confidence of the evidence were analyzed using the five considerations proposed by the GRADEpro GDT software (FP7-HEALTH.2010.3.1-1-two stage), which included study limitations (risk of bias), consistency of effect, imprecision, indirectness, and publication bias.

To gather information on the anxiolytic effects of gabapentin and pregabalin in cats, papers describing the effects, efficacy, and pharmacodynamics of both drugs were considered. Numerical rating scales were also used as an analytical tool to evaluate stress, fear, and anxiety in cats [3,11,18,19]. These tools are numerical scoring instruments based on the cat’s behavior during transportation to and from a veterinarian or medical consultation. These evaluation scores discriminate against the effects of gabapentinoids in domestic felines. To identify variations in behavior, the metrics used in the evaluation scores focused on the particular behavioral measurement that was applied. Metrics were based on objective observation to document a response (e.g., ease of physical examination, resistance during handling, tremors, salivation, vocalization, type of behavior—friendly/defensive). Likewise, guidelines for cat-friendly veterinary interaction were considered with the aim of understanding, interpreting, and appropriately responding to cats’ emotional states when pharmacotherapy may be an option to significantly reduce the cat’s protective emotional bias and the consequent risk of distress [4].

Information regarding the analgesic effect of gabapentinoids was defined using numerical rating scales with species-specific approaches such as the multidimensional scales of the UNESP-Botucatu Multidimensional Composite Pain Scale (UNESP-Botucatu MCPS), Glasgow Feline Composite Measure Pain Scale (CMPS-Feline), and Feline Grimace Scale (FGS) [2,20]. These scales assess physiological changes, including objective measurements of heart rate, respiratory rate, blood pressure, pupil size, facial expressions, and analysis of neuroendocrine parameters. Multidimensional numerical rating scales recognize the pain type (acute or chronic, abdominal, postoperative) and behavioral modifications, including posture, physical activity, vocalization, interaction with the environment and observers, and signs of fear, stress, and anxiety related to pain. Eligibility criteria also included manuscripts detailing the mechanism of action and dosage of gabapentinoids in pain management.

### 2.5. Collection and Evaluation of Additional Data

The data analyzed included author, year, source of publication, the reason for which the cat received gabapentin or pregabalin (e.g., during transport and visits to veterinary centers), animals with a history of anxiety or pain, the drug chosen, dose, route of administration, and the type of tool or instrument. Experimental studies were classified as randomized controlled trials, controlled clinical trials, and clinical trials that adopted a pre- and postoperative approach, as well as those that used a postoperative evaluation design. Observational studies were categorized as cohort studies, case–control studies, cross-sectional studies, and case series. Narrative reviews were also considered.

## 3. Results

**Figure 1:**
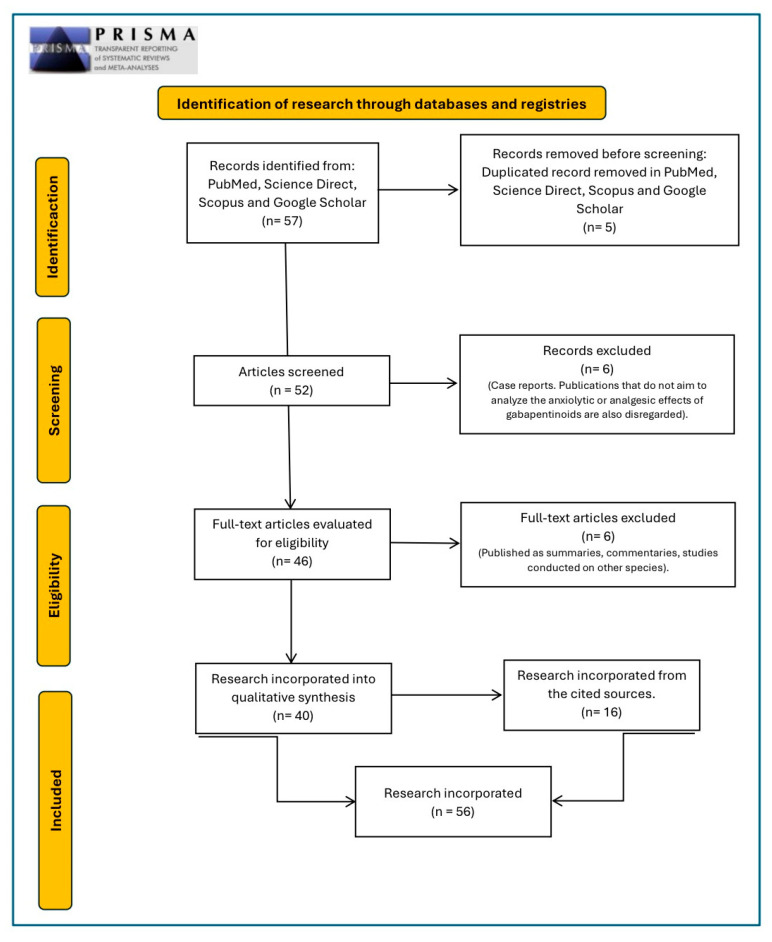
The PRISMA (guidelines, 2020) flow diagram illustrates the included and excluded studies, as well as the reasons for their exclusion (www.prisma-statement.org, accessed on 20 February 2025).

Following the inclusion and exclusion criteria according to the PRISMA guidelines, 57 articles were found. Five were duplicates and were discarded, and six were eliminated for not following the specific methodologies. Another six studies were excluded because they were clinical case reports in which pregabalin or gabapentin were mentioned solely as part of a treatment without an analysis of their outcomes. Therefore, 40 articles were selected (Figure 1).

### 3.1. Use of Gabapentin and Pregabalin in the Control and Treatment of Anxiety in Cats

Regarding the use of gabapentinoids for the management and treatment of stress, fear, or anxiety, 40 studies were reviewed. Furthermore, 26 studies refer to their use as anxiolytics, while 21 validate the use of gabapentin as a treatment for stress, fear, or anxiety in cats. Specific situations have been proposed for its prescription; six (28%) studies suggest its use for the management of cats in specific medical examinations (neurological, ophthalmological, cardiovascular, or dermatological examinations) [21,22,23,24,25,26]. The main use is to reduce anxiety in felines when they attend a veterinary clinic or hospital for specialty tests, where stress could complicate diagnosis due to altered results. Some actions, such as restraint, rapid or sudden movements, and loud sounds, can be intimidating and cause negative reactions in felines.

Nine studies (42%) report that gabapentin is a pharmacological option to reduce stress during veterinary consultations, improving animal handling. Stressors exert cumulative effects, meaning that an animal’s stress response is significantly intensified when faced with multiple stressors simultaneously, compared to exposure to a single stressor. This phenomenon is called “stressor stacking” or “trigger stacking” [27]. This drug has been shown to reduce stress and fear in cats during veterinary consultations, controlling fear-induced responses such as aggressive or resistant behaviors [12,18,19,21,28,29,30,31,32]. Furthermore, the anxiolytic results of gabapentin are validated by the application of numerical evaluation scores that highlight its efficacy as an anxiolytic [12,21,28,29,31,33]. This drug is also used to manage transport stress generated, where four (19%) of the publications used gabapentin with favorable results recorded in the evaluation scales applied to both guardians and veterinarians. Therefore, these findings are related to environmental changes, such as territorial displacement, in which the cat develops a lack of control and predictability [4,18,27,28].

These conditions can develop negative stress responses in cats, resulting in defensive or aggressive behaviors during different settings such as spay and neuter campaigns (orchiectomy and ovariohysterectomy), capture of feral cats, and within shelters or adoption centers. From the final search, five (23%) publications refer to the use of gabapentin to control fear and stress under these conditions. This drug manages to minimize negative affective states, avoiding health and welfare consequences of cats [31,33,34,35,36]. Due to its anxiolytic properties, gabapentin has also been used to reduce feline stress, fear, or anxiety in the perioperative period and hospitalization to reduce respiratory and hemodynamic alterations during the recovery. Three (14%) publications investigated these events and found that sedation scores increase, highlighting the fact that the cats’ anticipated stress and anxiety before surgery may have a greater impact on the hypothalamic–pituitary–adrenal axis and the sympathetic nervous system, promoting the perception of nociception and pain [11,32,36].

In comparison, pregabalin is an effective and safe anxiolytic that minimizes perceived threats in the physical and social environment, which is an essential component of cats’ emotional health. Furthermore, the most recent research reported that 59% of cats showed signs of distress during transport and 66% during veterinary visits. In this regard, five (100%) publications describe its usefulness as a therapy for controlling anxiety during transport and veterinary consultations [3,37,38,39,40].

### 3.2. Use of Gabapentin and Pregabalin in Pain Control and Treatment in Cats

Pain has a significant impact on a cat’s physical and emotional health, consequently affecting their welfare. In the analysis of this criterion, 12 articles used gabapentin as an analgesic; seven (58%) used it to treat acute pain, and five (42%) for chronic pain. The papers suggesting its use report its effectiveness as a treatment for non-surgical acute pain, as a perioperative coadjuvant analgesic during ovariohysterectomy, and as an analgesic in experimental models. This drug is also included in guidelines for feline pain management [2,10,14,15,41,42,43]. On the other hand, in the treatment of chronic pain, five publications address its use to manage maladaptive/neuropathic pain, as osteoarthritis treatment, and as adjuvant therapy in combination with non-steroidal analgesics (NAIDS) [44,45,46,47,48]. In contrast, two papers refer to pregabalin as an analgesic to treat maladaptive/neuropathic pain, hyperalgesia and allodynia, where its use can improve the effect of other analgesics [49,50]. Table 1 presents the analysis of the clinical trials considered in the systematic review.

## 4. Discussion

This systematic review identified and evaluated the use of gabapentin and pregabalin to manage stress, fear, and anxiety in domestic felines, as well as their use as adjuvants in the treatment of acute and chronic pain. This review offers a comprehensive approach that has allowed us to identify, select, and analyze the most current and relevant literature on the administration of gabapentin and pregabalin in cats, following the guidelines established for Systematic Reviews of Interventions, in accordance with the PRISMA statement. In this sense, stress is an involuntary reaction to tissue damage that can be expressed through alterations in the autonomic, metabolic, hormonal, immunological, and neuroendocrine systems [10]. In cats, several environmental changes and interspecific/intraspecific interactions can cause stress. These factors have cumulative effects when present in series or together, increasing the stress response and leading to a state of “stressor accumulation” [27].

Common causes of anxiety in cats are transportation and visits to veterinary centers. In these settings, factors such as unusual noises, strange smells, the presence of people and animals, and stimuli that are uncomfortable or painful disrupt their usual routine and might cause anxiety and stress. These stressors can be classified as physiological, those that arise due to transportation, medical consultation, or are caused by illness or trauma, hospitalization, or surgical procedures. Regardless of the cause, the stress response is a natural reaction so the animal can adapt and adequately respond to disturbances in their homeostasis. However, this same response can lead to negative physical and emotional effects [27,36]. The findings of the present systematic review highlight that, during veterinary care, cats may be subjected to surgical anesthetic procedures or hospitalization, implicating the separation from their owner, which can be associated with a greater number of cumulative effects and surgical stress. These events are a biological reaction that puts the animals’ homeostasis at risk [51]. These situations trigger specific and compensatory responses to prevent additional injuries, optimizing the availability of the substrates necessary for the proper functioning of vital organs [10,52].

The present review highlights the authors’ agreement that stress, fear, or anxiety can cause alterations in the GABAergic system, whether due to environmental factors or the experience of pain. These modifications can differ between different brain areas and are associated with several emotional responses observed during episodes of acute stress. In particular, the GABAergic system in brain regions such as the amygdala and hippocampus is critical for modulating the stress response [11,15,44].

Cats have unique pain-related behaviors, as they can develop new behaviors and lose previous ones. Therefore, it is crucial to develop an effective analgesic plan based on two fundamental principles: preventive analgesia and multimodal analgesia with the use of different drugs, including analgesic adjuvants such as gabapentinoids [14,20,48].

This review also highlights that gabapentin and pregabalin have a similar structure to the neurotransmitter GABA. However, they do not bind to GABA receptors and, therefore, are not classified as GABA agonists. Their mechanism of action has a selective binding to the α2δ−1 subunit of the calcium channel (N type), which is found in the mammalian central nervous system (CNS). This interaction inhibits calcium flow into neurons, resulting in a decrease in the release of monoamine neurotransmitters, such as norepinephrine, as well as a reduction in the synaptic transmission of excitatory neurotransmitters, including glutamate. These drugs also block calcium flow in the presynaptic terminal, which hinders the anterograde propagation of this process through the cytoplasm of the axon. Additionally, it has been observed that gabapentin promotes GABA synthesis, although there are other mechanisms that, through interaction with N-methyl-D-aspartate (NMDA) receptors and transient receptor potential (TRPV) channels, are attributed to the reduction in neuropathic pain. The modulation of noradrenergic receptors in the CNS, as well as the influence of cytokines and protein kinase C, also play an important role in the manifestation of these effects [16,27,28,29,32,43,44,46,53].

Both gabapentin and pregabalin act on neurobiological pain pathways by inhibiting the release of the excitatory neurotransmitter glutamate. Thus, both drugs are effective and well tolerated in the treatment of neuropathic pain, fibromyalgia, peripheral neuropathy, and anxiety and stress disorders in humans. For example, studies conducted in cats reported that pregabalin can be used to control and treat neuropathic pain due to its rapid absorption, linear pharmacokinetics, and high oral bioavailability (over 90%), while gabapentin’s bioavailability is 34–60% [54]. Furthermore, a mild, dose-dependent sedative effect has also been observed. For this reason, higher-dose treatment (3.95 mg/kg BID) has been proposed, resulting in greater potency compared to gabapentin, which requires higher doses and a higher frequency of administration (5–25 mg/kg BID–QID). The elimination half-life of gabapentin is between 5.0 and 7.0 h, while pregabalin has a half-life of 6.3 h. These estimates are comparable for both drugs, suggesting that they reach steady state within 24 to 48 h. Therefore, the recommended dosing frequency for both medications is 8 to 12 h [28,43,45,50,54].

The selected papers mention the efficacy of both substances, demonstrating the therapeutic potential for the treatment of stress, fear, and anxiety related or not to acute and chronic pain. Currently, objective evaluation tools for the efficacy of both drugs have been proposed, where the results show that cats receiving these anxiolytics during transportation obtained “excellent” or “good” categories. Consequently, veterinarians were 3–4 times more likely (*p* < 0.01) to easily perform the clinical examination after pre-medication (90 min before) than when using placebos [3,11,22,38].

In this sense, gabapentinoids have been commonly suggested for the treatment of chronic and neuropathic pain in companion animals, where they have proven effective for such purposes; however, it is also imperative to consider the control of acute pain. Focused research has used gabapentin for the management of acute perioperative and post-ovariohysterectomy pain in analgesic plans that may include the combination of this drug with tramadol, meloxicam, or buprenorphine [2,14,16]. Moreover, they have shown benefits in faster and more stable anesthetic recoveries. Following the evidence of their results, several authors have suggested the administration of gabapentin in soft tissue and orthopedic surgeries with promising results. Thus, both gabapentin and pregabalin are drugs with solid evidence to be considered therapeutic options for the management of acute and chronic pain, stress, fear, and anxiety in cats in different scenarios to which this species could be exposed. Based on the analysis of these results, it is suggested to expand the field of research into stress and pain control in cats in different models, as well as the need to conduct randomized, blinded, placebo-controlled trials that contribute to the current information [16,33,55].

In veterinary medicine, cats represent a significant challenge as their emotional states can be intensified by fear, stress, anxiety, pain, or frustration, which can trigger responses that generate cumulative stress-related effects. In this species, defensive behaviors often result from previous negative experiences, leading to reactions such as hissing, hitting, scratching, or biting, especially if they are handled roughly, preventing the cat from having the opportunity to flee or hide [4,18,30,32,56]. This review offers pharmacological alternatives that are considered effective strategies, along with adequate preparation before the visit and the implementation of careful and positive interactions. This aims to improve cats’ responses to unfamiliar situations, preventing them from becoming increasingly difficult to manage with each veterinary visit or in circumstances where they may experience acute or chronic pain. Likewise, through this systematic review of the existing literature, veterinarians can identify areas with a paucity of evidence (such as acute pain management), which also facilitates establishing the current state of knowledge regarding gabapentinoids in cats, a crucial aspect for developing new research projects, writing proposals, and promoting scientific evidence. Some perspectives that can be considered in the future in the management of acute and chronic pain in domestic cats are: conducting studies where the owners participate, especially evaluating the quality of life of animals. Also, further investigating behavioral studies where gabapentinoids can be evaluated as a factor that can modify behavior and facial expression. In addition to developing further studies that evaluate changes in physiological parameters induced by gabapentinoids, in order to establish a clinical correlation between various methods to ensure a more comprehensive assessment and management of acute and chronic pain.

Finally, the limitations of the current study include that the clinical trials considered do not use the same tools to validate drug efficacy, nor are the conditions the same. As with other systematic reviews, factors such as the inclusion of primary studies with greater bias or insufficient methodological quality, duplication and redundancy of experimental studies, and clinical or statistical heterogeneity, or situations that were sought to be controlled for with the inclusion and exclusion criteria, should also be considered.

## 5. Conclusions

Currently, the proper management of stress, fear, or anxiety, as well as the control and treatment of acute and chronic pain in cats through pharmacotherapy, is essential for feline clinical practice. The studies included in this systematic review present solid scientific evidence of the benefits of administering gabapentin and pregabalin in this species, promoting comfort and preventing the activation of the hypothalamic–pituitary–adrenal axis with its subsequent consequences on the welfare of domestic felines at different clinical settings, which were analyzed in this manuscript. Additional randomized, controlled clinical trials regarding the use of gabapentinoids are needed to complement the current knowledge about these drugs and validate these pharmacological alternatives for the benefit of cats and clinical practice.

## Figures and Tables

**Table 1:** Clinical trials were considered in the systematic review.

Author, Year	Study Design	Pharmacological Action	Stress, Anxiety, or Pain Measuring Instrument(s)	Dose	Certainty	Conclusions of the Study
GABAPENTIN						
**Azevedo, et al., 2023** [25]	Prospective, randomized, and blinded study	Anxiolytic (neurological examination)	NE	100 mg/cat	⊕⊕⊕⊕	Reduced stress, fear, and anxiety Facilitated the neurological examination
**Crowe, et al., 2022** [22]	Crossover, randomized, controlled, and blinded study	Anxiolytic (neurologic examination)	CSS	100 mg/cat	⊕⊕⊕⊕	Reduced stress Increased sedation Did not affect the pupillary diameter and the Schirmer test 1
**DuPont, et al., 2024** [26]	Prospective, double-blind, closed clinical trial	Anxiolytic (Neurologic examination)	CSS	100 mg/cat	⊕⊕⊕⊕	Anxiolytic Increased sedation in geriatric cats
**Eagan, et al., 2023** [34]	Randomized, blinded, and prospective study	Anxiolytic	CSS LTE VR	10 mg/kg	⊕⊕⊕⊕	Decreased stress in shelter cats Behavioral changes
**Gurney and Gower 2022** [18]	Prospective clinical trial	Anxiolytic	NE	20 mg/kg	⊕⊕⊕	In cats with hyperthyroidism, decreased transport stress and facilitated animal handling during consultation
**Van Haaften, et al., 2017** [28]	Randomized clinical trial	Anxiolytic	CSS VR CS	100 mg/cat	⊕⊕⊕⊕	Reduced stress and aggression Increased compliance during transportation, resulting in safe and effective treatment
**Hudec and Griffin 2020** [21]	Crossover, prospective, and blinded study	Anxiolytic	ECS OA G C	25–30.5 mg/cat	⊕⊕⊕⊕	Did not reduce serum cortisol/glucose concentrations Lower stress scores when measured by veterinarians and caregivers
**Kruszka, et al., 2021** [29]	Clinical, crossover, double-blind, randomized, and controlled	Anxiolytic Aggression management	CS	100 mg/cat (<7 kg) and 200 mg/cat (≥7 kg)	⊕⊕⊕⊕	Improves compliance during physical examination
**De Lombaert, et al., 2023** [19]	Experimental, prospective, randomized, and controlled study	Anxiolytic	CS CSS	100 mg/cat	⊕	Did not affect blood pressure Lower evaluation score, but without statistically significant differences
**Papageorgiou, et al., 2024** [11]	Prospective, blinded, controlled clinical trial	Anxiolytic	CSS VSS EAS	100 mg/cat	⊕	There were no differences between treatment (gabapentin/alprazolam) Lower CSS scores Increased sedation scores
**Papageorgiou, et al., 2024** [36]	Prospective, blinded, controlled clinical trial	Anxiolytic	CSS VSS GCMPS	100 mg/cat	⊕⊕⊕⊕	Gabapentin/alprazolam are effective anxiolytics during the postsurgical hospitalization
**Pankratz, et al., 2018** [33]	Double-blind, randomized, controlled study	Anxiolytic	CSS GSS	50 and 100 mg/cat	⊕⊕⊕⊕	Both doses reduced stress in confined cats without sedation
**Spano, et al., 2023** [31]	Double-blind, controlled, and randomized	Anxiolytic	CSS GSS EHS	5–30 mg/kg	⊕⊕⊕⊕	Reduced fear and stress during medical management in shelter cats
**Ruviaro, et al., 2022** [23]	Randomized, double-blind, controlled, prospective trial	Anxiolytic	CS GSS	100 mg/cat	⊕⊕⊕⊕	Effectively increased compliance without altering cardiovascular parameters
**Veronezi, et al., 2022** [24]	Prospective, randomized, blinded study	Anxiolytic	NE	100 mg/cat	⊕⊕⊕	Reduced anxiety without physiological or hemodynamic alterations
**Versteg, et al., 2024** [32]	Randomized, double-blind clinical trial	Anxiolytic	C	50–100 mg/cat	⊕	Gabapentin and integrative practices reduced stress and cortisol levels
**Jafarbeglou and Arkan 2025** [35]	Retrospective and blinded study	Anxiolytic	NE	100 mg/cat	⊕	An anxiolytic that improves sedation Fewer animals received rescue analgesia
**Raucourt and Masson 2025** [12]	Prospective, double-blind, randomized, crossover study	Anxiolytic	NRSAFE	100 mg/cat	⊕⊕⊕⊕	Supports early integration into behavioral therapy protocols Reduced fear and increased welfare Prevented phobias
**Rabbani, et al., 2021** [16]	Experimental, prospective, randomized study	Acute pain	GCMPS DIVAS	10 mg/cat	⊕⊕⊕⊕	The combination of gabapentin/tramadol improved analgesia and increased the mechanical nociceptive threshold
**Slovak and Costa 2021** [42]	Randomized study	Acute pain	GCMPS UNESP—Botucatu	10 mg/kg	⊕⊕	The transdermal route may be suitable Scores decreased Preliminary study with a small number of study animals
**Steagall, et al., 2018** [14]	Prospective, blinded, randomized, controlled study	Acute pain	GCMPS UNESP—Botucatu DIVAS	50 mg/cat	⊕⊕⊕	No differences between groups Gabapentin in combination with opioids is an alternative for perioperative pain
**Guedes, et al., 2018** [46]	Randomized, controlled, crossover study	Chronic pain	CSOM Omnidirectional piezoelectric accelerometer	10 mg/kg	⊕⊕⊕	Improved averages of tutors’ informational outcomes Decreased signs of pain
PREGABALIN						
**Lamminen, et al., 2021** [37]	Randomized, blinded, controlled study	Anxiolytic	VR RSDAF Ethogram (external observer)	5 and 10 mg/kg	⊕⊕⊕⊕	Reduces signs of anxiety and fear associated with traveling by car
**Lamminen, et al., 2023** [3]	Randomized, double-blind, placebo-controlled, parallel-group, multicenter study	Anxiolytic	NRST NRSCE VR	5 mg/kg	⊕⊕⊕⊕	Relief from anxiety and fear related to transportation and veterinary visits Improved medical management and feline welfare
**Li, et al., 2024** [40]	Randomized, blinded, crossover trial	Anxiolytic Sedative	The 13—point sedation scores	2.5, 5 and 10 mg/kg	⊕⊕⊕⊕	Causes sedation and anxiolytic effects The effects are dose-dependent.

CS = Compliance Score; CSS = Cat stress score; GSS = Global sedation score; C = serum cortisol; ECS = Ease-Of-Catheterization Scale; EAS = Enclosure activity score; EHS = Ease of handling score; RSDAF = Rating of the signs of distress, anxiety and/or fear by the cat tutor; VSS = Volpato sedation score; G = Serum glucose; LTE = Latency to emerge from hiding; NE = not specified; OA = Owner assessment; GCMPS = Measurement Glasgow; UNESP-Botucatu = Multidimensional composite Feline pain scale; DIVAS = Dynamic interactive visual analog scale; CSOM = Client-specific outcome measure; NRST = Numerical rating score for the owner’s assessment of the treatment effect based of the cat’s stress, anxiety and/or fear during the transportation in a car; NRSCE = Numerical rating score for the owner’s assessment of the treatment effect based of the cat’s stress, anxiety and/or fear during the clinical examination at the clinic; NRSAFE = Numerical rating scale to asses the fear score during the clinical examination; VR = Video recording. Certainty assessment: High = ⊕⊕⊕⊕ Moderate = ⊕⊕⊕ Low = ⊕⊕ Very low = ⊕.

## Data Availability

The original contributions presented in the study are included in the article, further inquiries can be directed to the corresponding author.
